# Prevalence and correlates of low-level viremia and viral load non-suppression among adults on HIV treatment: Results from the Tanzania HIV Impact Survey, 2022–2023

**DOI:** 10.1371/journal.pone.0344439

**Published:** 2026-03-26

**Authors:** Samwel Sumba, Alexander Kailembo, Abbas Ismail, Prosper Njau, Alice Wang, Geofrey Mchau, Baraka Revocatus, Eliezer Anthony Taluka, Mary Mayige, Tepa Nkumbula, Faki Haji Faki, Optatus Malewo, Nyambura Moremi, Emilian Karugendo, Fahima Issa, Ame Masemo, Rebecca Laws, Divya Patel, Haruka Maruyama, Jerome Kamwela, Wafaa El-Sadr, George S. Mgomella, Mahesh Swaminathan, Sarah Porter, Ahmed Khatib

**Affiliations:** 1 Tanzania Commission for AIDS, Dodoma Tanzania; 2 U.S. Centers for Diseases Control and Prevention, Division of Global HIV and TB, Dar es Salaam, Tanzania; 3 National AIDS, STIs and Hepatitis Control Programme, Dodoma, Tanzania; 4 Bugando Medical Centre, Mwanza, Tanzania; 5 ICAP at Columbia University, Dar es Salaam, Tanzania; 6 National Institute of Medical Research, Dar es Salaam, Tanzania; 7 Zanzibar Integrated HIV, Hepatitis, Tuberculosis, and Leprosy Programme, Unguja, Zanzibar; 8 National Public Health Laboratory, Dar es Salaam, Tanzania; 9 National Bureau of Statistics, Dodoma, Tanzania; 10 Office of Chief Government Statistician, Unguja, Zanzibar; 11 Zanzibar Health Research Institute, Unguja, Zanzibar; 12 U.S. Centers for Diseases Control and Prevention, Division of Global HIV and TB, Atlanta, United States of America; 13 ICAP at Columbia University, New York, United States of America; 14 Zanzibar AIDS Commission, Unguja, Zanzibar; Institute of Tropical Medicine / University of Antwerp, BELGIUM

## Abstract

**Background:**

While HIV viral load (VL) testing remained a critical approach for monitoring antiretroviral therapy (ART) effectiveness among people living with HIV (PLHIV), limited national data existed on the magnitude and factors associated with key VL thresholds, including low-level viremia (LLV) and VL non-suppression in Tanzania. We analyzed the prevalence and socio-demographic and behavioral factors associated with LLV and VL non-suppression among PLHIV on ART participating in a nationally representative survey.

**Methods:**

We analyzed data from the Tanzania HIV Impact Survey (THIS) 2022–2023 among PLHIV aged 15 years and older. The analysis included 1,485 PLHIV on ART with VL results. Laboratory-based testing was conducted for qualitative detection of antiretroviral (ARV) drugs and quantitative evaluation of VL. Three VL levels were computed for the analyses: undetectable VL (<50 copies/mL); LLV (50–999 copies/mL); and VL non-suppression (≥1000 copies/mL). Unweighted absolute numbers and weighted percentages were reported for descriptive analysis. We used modified Poisson regression models to examine separately the prevalence and factors associated with LLV and VL non-suppression. We reported adjusted prevalence ratios (aPR), 95% confidence intervals (CIs) and p-values <0.05 were considered statistically significant.

**Results:**

Overall, 76% of PLHIV in Tanzania had undetectable VL, 18% had LLV, and 6% had VL non-suppression. Among PLHIV with either LLV or undetectable VL, 19.5% had LLV and after multivariable adjustment, absence of ARV drugs detected in blood was the only factor significantly associated with LLV. Additionally, among PLHIV with either VL non-suppression or undetectable VL, 7.2% had VL non-suppression and after multivariable adjustment, absence of ARV drugs detected in blood and alcohol use were associated with VL non-suppression.

**Conclusion:**

In this nationally representative analysis of PLHIV in Tanzania, most people had undetectable viral load, while LLV was common and VL non-suppression was less frequent. Absence of ARV drugs detected in blood was associated with both LLV and VL non-suppression, underscoring the importance of recent ART exposure. These findings suggested that LLV warranted programmatic attention alongside VL non-suppression to support sustained viral suppression.

## Introduction

To end the HIV epidemic as a public health threat by 2030, the UNAIDS 95-95-95 targets aimed for 95% of all people living with HIV (PLHIV) to know their HIV status, 95% of all PLHIV who know their status to receive antiretroviral therapy (ART), and 95% of all people receiving ART to achieve viral load (VL) suppression by 2030 [1]. In addition to routine HIV programmatic monitoring, national surveys like Tanzania HIV Impact Survey (THIS) 2022–2023, part of the global Population-based HIV Impact Assessment (PHIA) project, played a crucial role in evaluating progress towards achieving the UNAIDS targets and advancing HIV epidemic control in Tanzania [2]. According to THIS 2022–2023, HIV prevalence among adults was 4.4%, corresponding to approximately 1.55 million PLHIV [3]. Additionally, 94.3% of PLHIV on ART had achieved VL suppression [3], highlighting significant progress in the national HIV response. However, challenges remained, particularly with PLHIV on ART who, despite treatment, continue to pose a risk for HIV transmission and for poor clinical outcomes due to suboptimal VL suppression.

There were two groups of PLHIV with suboptimal VL suppression: those classified as VL non-suppression, who pose a risk for HIV transmission, and those with low-level viremia (LLV), who have a lower risk of transmission. A third category included PLHIV with undetectable VL, who were considered to have zero risk of transmission. These groups were consistent with World Health Organization (WHO) guidelines on the three key categories for HIV VL measurements and risk of transmission [4]. Although PLHIV with LLV were considered to have a negligible risk of transmission, this risk was not zero. Also, LLV may have served as an early indicator of VL non-suppression [5]. Additionally, the clinical significance of LLV may have reflected early signs of treatment failure or adherence challenges and remained an area of ongoing interest and observation [4,6,7]. Therefore, monitoring LLV was increasingly critical, as both VL non-suppression and LLV could have signalled potential issues with treatment adherence and effectiveness [5].

While WHO recommended targeted interventions for PLHIV with both VL non-suppression and LLV [5], many countries including Tanzania, had established guidelines for VL non-suppression but had yet to implement specific interventions for LLV. Existing evidence showed that VL non-suppression is associated with several demographic and socioeconomic factors, including male sex, younger age, lower educational level, lower household income, and lower relative wealth [8–11]. Emerging studies suggested that LLV shared many of these same risk factors [12,13]. As such, generating robust data on both VL non-suppression and LLV was critical to identifying at-risk populations and informing targeted interventions to improve treatment outcomes and reduce HIV transmission.

Although Tanzania was expected to incorporate WHO recommendations on LLV into national guidelines and introduce intervention strategies for both VL non-suppression and LLV, nationally representative data on burden and associated factors remained limited. Using data from THIS 2022–2023, we assessed the prevalence of LLV and VL non-suppression and examined the socio-demographic and behavioral correlates among PLHIV on ART in Tanzania.

## Methods

### Survey design and procedures

We used nationally representative data from THIS 2022–2023. This cross-sectional survey used a two-stage, stratified cluster design to obtain a nationally-representative sample of adults 15 years and older in Tanzania. It included a questionnaire administered through a face-to-face interview, a point-of-care rapid HIV test, and blood sample collection for additional biomarker testing. Final HIV status was determined through the national testing algorithm. For confirmed HIV-positive samples, laboratory-based testing was conducted for quantitative evaluation of VL and qualitative detection of ARV (dolutegravir, efavirenz, atazanavir, and lopinavir). A full description of the survey methods, including specific procedures on VL testing and detection of ARV, had been published elsewhere [3].

### Study population

THIS 2022–2023 included 33,663 participants who were interviewed and had a final HIV status. Of those interviewed, 1,850 individuals were confirmed to be HIV positive. PLHIV were classified as being on ART if they had a detectable antiretroviral in their blood, self-reported being on ART, or both. The survey identified 1,555 PLHIV on ART. VL results were available for all 1,555 PLHIV [3]. Seventy PLHIV were excluded due to missing data on independent variables. Therefore, the final analysis included 1,485 PLHIV on ART with VL results.

### Study variables

The primary outcomes of interest were LLV and VL non-suppression, which were analyzed separately. The independent variables were sociodemographic and behavioral variables.

#### Outcome variables.

To generate the two outcome variables, three VL measurement categories were first defined as follows: PLHIV with HIV RNA copies <50 per milliliter (<50 copies/mL) (undetectable VL); PLHIV with 50–999 copies/mL (LLV); and PLHIV with ≥1000 copies/mL (VL non-suppression). Therefore, the first outcome variable was a binary variable derived from PLHIV with LLV compared to those with undetectable VL, while the second outcome variable was a binary variable derived from PLHIV with VL non-suppression compared to those with undetectable VL.

#### Independent variables.

The factors evaluated for association with LLV and VL non-suppression included sex, age group in years (15–24, 25–34, 35–49, and ≥50 years), residence (urban, rural), marital status (never married, married/living together, separated/divorced, widowed), wealth quintile (wealth variable was constructed using standard methods for household surveys [14]), educational attainment (no education, primary education, secondary education or higher), knowledge of HIV (good, poor), discriminatory attitude (yes, no), alcohol use (yes, no), tobacco smoking (yes, no), and detection of ARV drugs (yes, no).

Knowledge of HIV was assessed by asking participants to either agree or disagree with the following two statements: (I) “When taken as prescribed by a health worker, HIV medications decrease the amount of HIV in the blood of people living with HIV. Therefore, the amount of virus in their blood becomes too low to detect in a laboratory test”; and (II) “A person living with HIV who is taking HIV medications cannot pass HIV to a sexual partner once a laboratory test can no longer detect the HIV virus in their blood”. Knowledge of HIV was analyzed as a dichotomous variable; good knowledge (agreed with both statements) and poor knowledge (disagreed with either statement or with both) [3].

Discriminatory attitude was assessed using the following two questions: (I) “Would you buy fresh vegetables from a shopkeeper or vendor if you knew that this person had HIV?”; and (II) “Do you think that children living with HIV should be able to attend school with children who are HIV negative?”. Discriminatory attitude was analyzed as a dichotomous variable; no discriminatory attitude (answered “yes” to both questions) and discriminatory attitude (answered “no” to either question or both questions) [3].

The alcohol use dichotomous variable was obtained from the question “In the last 30 days, have you had any drinks containing alcohol?” The smoking tobacco variable was obtained from the question “Do you currently smoke any form of tobacco on a daily basis, less than daily, or not at all?” Smoking tobacco was analyzed as a dichotomous variable; smokers (reported “daily” or “less than daily” use) and non-smokers (reported “not at all”) [3].

Detection of ARV drugs in blood was operationalized as a binary variable (yes/no) based on qualitative laboratory screening of dried blood spot (DBS) specimens collected from all participants who tested HIV positive. DBS samples were analyzed for the presence of four ARV agents, dolutegravir, efavirenz, atazanavir, and lopinavir, selected as markers of the most commonly prescribed first and second-line ART regimens in Tanzania.

### Statistical analysis

Unweighted absolute numbers and weighted percentages were reported for descriptive analysis. For bivariable analysis, we reported absolute numbers, weighted percentages, and p-values from a chi-squared test to assess differences in LLV and VL non-suppression by independent variables. Any p-value <0.05 was considered statistically significant. In analytical models, multivariable analyses were conducted using modified Poisson regression models to examine separately the associations between the independent variables and LLV and VL non-suppression. All independent variables – sex, age, residence, marital status, wealth, education, HIV knowledge, discriminatory attitude, alcohol use, smoking tobacco, and ARV detection in blood – were included in the multivariable models, regardless of their statistical significance in bivariable analyses to account for biases associated with data-driven variable selection. Sampling weights to account for study design and non-response were used. The jackknife replication method was used for producing variance estimates. We reported adjusted prevalence ratios (aPR) and 95% confidence intervals (CIs). We used Stata version 17 (StataCorp, 2019) for all statistical analyses.

### Ethical approvals

THIS 2022–2023 protocol was approved by the National Institute for Medical Research, Tanzania; Zanzibar Health Research Institute, Zanzibar; U.S. CDC, Atlanta, GA, USA; and ICAP at Columbia University, New York, NY, USA. The survey was reviewed in accordance with CDC human research protection procedures and was determined to be research, but CDC investigators did not interact with human subjects or have access to identifiable data or specimens for research purposes. Prior to participation, verbal informed consent or assent was obtained from all participants, as approved by the overseeing Institutional Review Boards. Trained interviewers administered standardized consent script in the participant’s preferred language. Verbal consent was documented electronically at the time of the interview. All collected data were handled with approved ethical and data protection procedures.

## Results

### Characteristics of the study population

Among PLHIV on ART in Tanzania in this analysis, 68.3% were female, 75.5% were aged 35 years or older, and 51.0% were married or living with a partner. More than half (56.3%) resided in rural areas, and the majority (69.4%) had completed primary level education (Table 1).

**Table 1:** Characteristics of people living with HIV aged 15 years and older who were on antiretroviral therapy from the Tanzania HIV Impact Survey, 2022-2023.

Characteristic		n^#^	Weighted (%)^+^
Sex	Female	1,055	68.3
	Male	430	31.7
Age (years)	50+	505	32.2
	35-49	648	43.3
	25-34	260	18.4
	15-24	72	6.1
Residence	Urban	538	43.7
	Rural	947	56.3
Marital status	Married/living together	765	51.0
	Divorced/separated	310	20.9
	Widowed	269	16.4
	Never married	141	11.7
Wealth (quintiles)	Highest	170	15.3
	Fourth	305	22.4
	Middle	384	21.1
	Second	372	22.8
	Lowest	254	18.4
Education	Secondary and above	186	14.7
	Primary	1,058	69.4
	No education	241	15.9
HIV knowledge	Good	806	54.6
	Poor	679	45.4
Discriminatory attitude	No	1,344	90.7
	Yes	141	9.3
Alcohol use	No	988	69.8
	Yes	497	30.2
Smoking tobacco	No	1,350	89.8
	Yes	135	10.2
ARV detected in blood	Yes	1,419	95.4
	No	66	4.6

^#^absolute number of PLHIV on ART with VL results from the study population, THIS 2022–2023.

^+^weighted proportions of PLHIV on ART from Tanzania, THIS 2022–2023.

### Prevalence of LLV and associated factors

Among PLHIV with either LLV or undetectable VL, 19.5% had LLV and 80.5% had undetectable VL. In the multivariable analysis, absence of ARV detected in blood was the only factor significantly associated with LLV (Table 2).

**Table 2:** Prevalence of low-level viremia and multivariable analysis of associated factors among people living with HIV aged 15 years and older who were on antiretroviral therapy from the Tanzania HIV Impact Survey, 2022–2023.

	Prevalence			Multivariable^#^ model
	Low-level viremia	Undetectable viral load	p-value^+^	aPR^$^
	n (%)^*^	n (%)		
	283 (19.5)	1,118 (80.5)		
**Sex**				
Female	183 (16.8)	816 (83.2)	**0.0035**	Reference
Male	100 (25.4)	302 (74.6)		1.30 (0.95-1.78)
**Age (years)**				
50+	113 (21.8)	376 (78.2)	0.3734	Reference
35-49	118 (18.0)	499 (82.0)		0.85 (0.66-1.11)
25-34	39 (17.1)	196 (82.9)		0.82 (0.54-1.25)
15-24	13 (25.3)	47 (74.7)		1.26 (0.64-2.48)
**Residence**				
Urban	88 (18.3)	417 (81.7)	0.4781	Reference
Rural	195 (20.4)	701 (79.6)		1.04 (0.74-1.47)
**Marital status**				
Married/cohabiting	144 (19.9)	578 (80.1)	0.7714	Reference
Divorced/separated	63 (20.2)	233 (79.8)		1.06 (0.75-1.49)
Widowed	50 (16.5)	210 (83.5)		0.92 (0.62-1.34)
Never married	26 (20.6)	97 (79.4)		1.03 (0.62-1.71)
**Wealth (quintiles)**				
Highest	26 (17.5)	133 (82.5)	0.9394	Reference
Fourth	55 (18.6)	231 (81.4)		0.98 (0.56-1.72)
Middle	71 (20.0)	301 (80.0)		0.98 (0.57-1.66)
Second	76 (20.0)	275 (80.0)		0.97 (0.53-1.77)
Lowest	55 (21.1)	178 (78.9)		1.03 (0.62-1.72)
**Education**				
Secondary/above	29 (17.5)	142 (82.5)	0.7968	Reference
Primary	205 (19.9)	797 (80.1)		1.14 (0.71-1.83)
No education	49 (19.6)	179 (80.4)		1.14 (0.71-1.81)
**HIV knowledge**				
Good	160 (18.9)	609 (81.1)	0.6129	Reference
Poor	123 (20.2)	509 (79.8)		1.08 (0.81-1.44)
**Discriminatory attitude**				
No	249 (18.8)	1,025 (81.2)	0.1565	Reference
Yes	34 (26.2)	93 (73.8)		1.37 (0.88-2.13)
**Alcohol use**				
No	177 (17.9)	746 (82.1)	0.0760	Reference
Yes	106 (23.0)	372 (77.0)		1.13 (0.86-1.48)
**Smoking tobacco**				
No	247 (18.1)	1,028 (81.9)	**0.0025**	Reference
Yes	36 (32.1)	90 (67.9)		1.44 (0.99-2.07)
**ARV detected in blood**				
Yes	270 (19.0)	1,094 (81.0)	**0.0252**	**Reference**
No	13 (36.6)	24 (63.4)		**2.10 (1.24-3.55)**

# multivariable Poisson regression model adjusted for sex, age, residence, marital status, wealth, education, HIV knowledge, discriminatory attitude, alcohol use, smoking tobacco, and ARV detection.

+ p-values from a chi-squared test to assess differences in low-level viremia by independent variables.

$ adjusted prevalence ratio using Poisson regression model.

* absolute numbers and weighted proportions.

### Prevalence of VL non-suppression and associated factors

Among PLHIV with either VL non-suppression or undetectable VL, 7.2% had VL non-suppression and 92.8% achieved undetectable VL. In the multivariable analysis, VL non-suppression was 8.85 (95%CI: 5.13–15.29) times higher among PLHIV without ARV detected in blood compared to those with detectable ARV, and alcohol users were less likely to experience VL non-suppression compared to non-users (Table 3).

**Table 3:** Prevalence of viral load non-suppression and multivariable analysis of associated factors among people living with HIV aged 15 years and older who were on antiretroviral therapy from the Tanzania HIV Impact Survey, 2022-2023.

	Prevalence			Multivariable^#^ model
	Viral load non-suppression	Undetectable viral load	p-value^+^	aPR^$^
	n (%)^##^	n (%)		
	84 (7.2)	1,118 (92.8)		
**Sex**				
Female	56 (6.4)	816 (93.6)	0.1426	Reference
Male	28 (9.1)	302 (90.9)		1.31 (0.69-2.48)
**Age (years)**				
50+	16 (4.7)	376 (95.3)	**0.0013**	Reference
35-49	31 (5.5)	499 (94.5)		1.04 (0.53-2.06)
25-34	25 (11.6)	196 (88.4)		1.88 (0.96-3.70)
15-24	12 (18.6)	47 (81.4)		1.94 (0.60-6.21)
**Residence**				
Urban	33 (7.5)	417 (92.5)	0.7888	Reference
Rural	51 (7.0)	701 (93.0)		1.01 (0.44-2.36)
**Marital status**				
Married/cohabiting	43 (7.2)	578 (92.8)	**0.0321**	Reference
Divorced/separated	14 (5.5)	233 (94.5)		0.92 (0.46-1.83)
Widowed	9 (4.7)	210 (95.3)		1.14 (0.49-2.64)
Never married	18 (13.7)	97 (86.3)		1.22 (0.60-2.46)
**Wealth (quintiles)**				
Highest	11 (7.5)	133 (92.5)	0.1447	Reference
Fourth	19 (7.1)	231 (92.9)		1.01 (0.45-2.27)
Middle	12 (3.3)	301 (96.7)		0.56 (0.19-1.62)
Second	21 (8.2)	275 (91.8)		1.05 (0.33-3.37)
Lowest	21 (10.2)	178 (89.8)		1.17 (0.32-4.31)
**Education**				
Secondary/above	15 (9.2)	142 (90.8)	0.5246	Reference
Primary	56 (7.0)	797 (93.0)		0.87 (0.49-1.55)
No education	13 (6.0)	179 (94.0)		0.66 (0.34-1.29)
**HIV knowledge**				
Good	37 (6.1)	609 (93.9)	0.1338	Reference
Poor	47 (8.5)	509 (91.5)		1.55 (0.99-2.43)
**Discriminatory attitude**				
No	70 (6.5)	1,025 (93.5)	**0.0161**	Reference
Yes	14 (14.7)	93 (85.3)		1.50 (0.78-2.89)
**Alcohol use**				
No	65 (8.4)	746 (91.6)	**0.0428**	Reference
Yes	19 (4.3)	372 (95.7)		**0.43 (0.25-0.74)** ^**^
**Smoking tobacco**				
No	75 (6.9)	1,028 (93.1)	0.2582	Reference
Yes	9 (10.2)	90 (89.8)		1.76 (0.90-3.46)
**ARV detected in blood**				
Yes	55 (5.0)	1,094 (95.0)	0.0000	Reference
No	29 (54.1)	24 (45.9)		**8.85 (5.13-15.29)** ^***^

^#^multivariable Poisson regression model adjusted for sex, age, residence, marital status, wealth, education, HIV knowledge, discriminatory attitude, alcohol use, smoking tobacco, ARV detection.

^+^p-values from a chi-squared test to assess differences in low-level viremia by independent variables.

^$^adjusted prevalence ratio using Poisson regression model.

^##^absolute numbers and weighted proportions.

* p-value <0.05, ** p-value <0.01, *** p-value <0.001.

## Discussion

Using nationally representative data from Tanzania, we observed that nearly one in five PLHIV on ART had LLV, indicating a substantial gap in achieving complete viral suppression. While the 2019 National HIV Guidelines recommended enhanced adherence counselling for PLHIV with VL non-suppression, they lacked specific guidance for PLHIV with LLV such as provision of enhanced adherence counselling and viral load monitoring [15]. These findings highlighted the urgency of integrating evidence-based LLV interventions into forthcoming guideline updates. Globally, LLV was increasingly recognized as an early marker of virologic failure, drug resistance, and non-AIDS-related conditions such as cardiovascular and renal disease [16–18]. Studies from Kenya and Nigeria supported LLV’s link to future treatment failure [19,20]. Clear communication on the risks of LLV and VL non-suppression, alongside efforts to sustain undetectable VL, was essential to improving outcomes and preventing HIV transmission [21].

Across analyses of both LLV and VL non-suppression, absence of ARV drugs was associated with poorer virologic outcomes, underscoring the importance of recent ART exposure in maintaining virologic control. This finding was consistent with prior evidence demonstrating that objective measures of ART exposure predicted viral suppression and outperformed self-reported adherence, which could be subject to biases [22–24]. Although the subgroup without ARV detected was relatively small, raising concerns about estimate precision, the consistency of this association across both outcomes supported the robustness of the observed relationship [25].

Our study did not find significant associations between LLV and other characteristics, consistent with previous research [26,27], and may reflect limited independent effects of these factors after adjustment for ART exposure. Nevertheless, LLV remained a valuable marker for detecting early treatment challenges, even in the absence of clear associations with demographic or behavioural characteristics. Furthermore, our study showed alcohol use was inversely associated with viral load non-suppression, contrary to prior evidence linking alcohol use to poorer adherence and virologic outcomes [28,29]. This finding may have reflected measurement limitations, reporting bias, or residual confounding and should be interpreted cautiously.

Additionally, in contrast to previous studies that reported associations between sex [30,31], lower education [8,10] and lower wealth [11,32] with VL non-suppression, we did not find significant associations between these characteristics and either LLV or VL non-suppression in this analysis. HIV knowledge was also not associated with LLV or VL non-suppression, however, a substantial proportion of adults receiving ART lacked adequate understanding of HIV treatment and viral suppression. This observation suggested potential gaps in knowledge, consistent with prior reports from sub-Saharan Africa, and indicated that continued emphasis on community-level health education and messaging, including undetectable = untransmittable (U = U) initiatives, may be warranted [33–35].

The strength of our study lay in the scale and design of THIS-2022–2023, a nationally representative, household-based survey aimed at measuring viral load suppression prevalence among the general adult population at both national and sub-national levels. However, our study has limitations. As a cross-sectional survey, THIS 2022–2023 could not establish causality. Self-reported responses were subject to social desirability biases or misremembering. Additionally, we could not differentiate the causes of LLV or viral load non-suppression among ART users as the HIV drug resistance data were not available in time for these paper and other relevant variables such as adherence questions were not included due to missing observations. Finally, some findings warranted cautious interpretation due to small sample sizes.

## Conclusion

This study provided important insights into the prevalence and associated factors of LLV and VL non-suppression among PLHIV on ART in Tanzania. Most PLHIV had undetectable viral load, while LLV was common and VL non-suppression was less frequent. Absence of ARV drugs detected in blood was associated with both LLV and VL non-suppression, underscoring the importance of recent ART exposure. These findings suggested that LLV warranted programmatic attention alongside VL non-suppression to support sustained viral suppression. Integrating targeted LLV monitoring and interventions into national guidelines was vital to achieving epidemic control.
